# Cost‐per‐diagnosis as a metric for monitoring cost‐effectiveness of HIV testing programmes in low‐income settings in southern Africa: health economic and modelling analysis

**DOI:** 10.1002/jia2.25325

**Published:** 2019-07-09

**Authors:** Andrew N Phillips, Valentina Cambiano, Fumiyo Nakagawa, Loveleen Bansi‐Matharu, David Wilson, Ilesh Jani, Tsitsi Apollo, Mark Sculpher, Timothy Hallett, Cliff Kerr, Joep J van Oosterhout, Jeffrey W Eaton, Janne Estill, Brian Williams, Naoko Doi, Frances Cowan, Olivia Keiser, Deborah Ford, Karin Hatzold, Ruanne Barnabas, Helen Ayles, Gesine Meyer‐Rath, Lisa Nelson, Cheryl Johnson, Rachel Baggaley, Ade Fakoya, Andreas Jahn, Paul Revill

**Affiliations:** ^1^ Institute for Global Health UCL London UK; ^2^ Burnet Institute Melbourne Australia; ^3^ National Institute of Health Maputo Mozambique; ^4^ Ministry of Health Zimbabwe, Harare Zimbabwe; ^5^ Centre for Health Economics University of York York UK; ^6^ Department of Infectious Disease Epidemiology Imperial College London London UK; ^7^ University of Sydney Sydney Australia; ^8^ Dignitas International Zomba Malawi; ^9^ College of Medicine Blantyre Malawi; ^10^ Institute of Global Health University of Geneva Geneva Switzerland; ^11^ Institute of Mathematical Statistics and Actuarial Science University of Bern Bern Switzerland; ^12^ SACEMA Stellenbosch University Stellenbosch South Africa; ^13^ Clinton Health Access Initiative (CHAI) NY USA; ^14^ CeSHHAR Harare Zimbabwe; ^15^ Liverpool School of Tropical Medicine Liverpool UK; ^16^ MRC Clinical Trials Unit at UCL UCL London UK; ^17^ PSI Harare Zimbabwe; ^18^ University of Washington Seattle WA USA; ^19^ ZAMBART Lusaka Zambia; ^20^ Health Economics and Epidemiology Research Office Department of Internal Medicine Faculty of Health Sciences University of the Witwatersrand Johannesburg South Africa; ^21^ Department for Global Health Boston University Boston MA USA; ^22^ CDC Uganda Kampala Uganda; ^23^ World Health Organisation Geneva Switzerland; ^24^ The Global Fund Geneva Switzerland; ^25^ Ministry of Health Lilongwe Malawi

**Keywords:** testing, HIV, cost‐effectiveness, modelling, health systems

## Abstract

**Introduction:**

As prevalence of undiagnosed HIV declines, it is unclear whether testing programmes will be cost‐effective. To guide their HIV testing programmes, countries require appropriate metrics that can be measured. The cost‐per‐diagnosis is potentially a useful metric.

**Methods:**

We simulated a series of setting‐scenarios for adult HIV epidemics and ART programmes typical of settings in southern Africa using an individual‐based model and projected forward from 2018 under two policies: (i) a minimum package of “core” testing (i.e. testing in pregnant women, for diagnosis of symptoms, in sex workers, and in men coming forward for circumcision) is conducted, and (ii) core‐testing as above plus additional testing beyond this (“additional‐testing”), for which we specify different rates of testing and various degrees to which those with HIV are more likely to test than those without HIV. We also considered a plausible range of unit test costs. The aim was to assess the relationship between cost‐per‐diagnosis and the incremental cost‐effectiveness ratio (ICER) of the additional‐testing policy. The discount rate used in the base case was 3% per annum (costs in 2018 U.S. dollars).

**Results:**

There was a strong graded relationship between the cost‐per‐diagnosis and the ICER. Overall, the ICER was below $500 per‐DALY‐averted (the cost‐effectiveness threshold used in primary analysis) so long as the cost‐per‐diagnosis was below $315. This threshold cost‐per‐diagnosis was similar according to epidemic and programmatic features including the prevalence of undiagnosed HIV, the HIV incidence and a measure of HIV programme quality (the proportion of HIV diagnosed people having a viral load <1000 copies/mL). However, restricting to women, additional‐testing did not appear cost‐effective even at a cost‐per‐diagnosis of below $50, while restricting to men additional‐testing was cost‐effective up to a cost‐per‐diagnosis of $585. The threshold cost per diagnosis for testing in men to be cost‐effective fell to $256 when the cost‐effectiveness threshold was $300 instead of $500, and to $81 when considering a discount rate of 10% per annum.

**Conclusions:**

For testing programmes in low‐income settings in southern African there is an extremely strong relationship between the cost‐per‐diagnosis and the cost‐per‐DALY averted, indicating that the cost‐per‐diagnosis can be used to monitor the cost‐effectiveness of testing programmes.

## 1 Introduction

A key strategy in global efforts to reduce incidence of HIV infection is to aim for high levels of diagnosis of HIV‐positive individuals and to help people with diagnosed HIV to initiate and remain on ART with high adherence^1^, ^2^, ^3^. Countries are being encouraged to set up large‐scale testing programmes to attain the target of at least 90% of people living with HIV (PLHIV) being diagnosed by 2020 and at least 95% by 2025. However, as the prevalence of undiagnosed HIV declines over time, the proportion of HIV tests that result in diagnosis of an HIV‐infected person will also decline. Thus, the cost‐effectiveness of HIV testing strategies becomes increasingly uncertain. Various HIV testing approaches have been used and they differ in the extent to which tests are more likely to be conducted in PLHIV than in those without, and in their cost per test conducted^4^, ^5^, ^6^. For example, testing programmes based on partner notification might have a high cost per HIV test but also a high test‐positive rate (sometimes referred to as “yield”). Some testing approaches that are currently implemented may not be cost‐effective. Countries require appropriate metrics that can be feasibly measured to guide their HIV testing programmes. A key metric of potential interest is the cost‐per‐diagnosis (i.e. diagnosis in persons never previously having had a positive test). However, it is not intuitively clear what is the maximum cost‐per‐diagnosis at which HIV testing in low‐income countries in southern Africa remains cost‐effective. Nor is it clear whether the value of this threshold depends substantially on epidemic or programmatic features. In this paper, we make use of simulation modelling of HIV epidemics and testing programmes to consider these questions to provide guidance to HIV testing programmes.

## 2 Methods

We used an individual‐based stochastic simulation model of HIV transmission, progression and the effect of ART in adult populations in southern Africa, which has been described previously 7, 8 and is detailed in supplementary materials. Each time the model is run it simulates data in three‐monthly time steps on whether the person has an ongoing primary condomless sex partner, the number of other condomless sex partners, HIV testing, HIV acquisition risk and, in people living with HIV, viral load, CD4 count, use of specific ART drugs, adherence, resistance and risk of HIV‐related death. In addition to the health benefit to the individual of being diagnosed earlier, the model provides a means by which the effects of testing on increasing the proportion of HIV‐positive people who are on ART translate into greater health for the individual and lower onward transmission, and of estimating the overall number of disability adjusted life years (DALYs) averted in the adult population as a whole.

We initially based the modelled scenarios on data from Malawi but varied parameter values (i.e. we independently sampled randomly from the distributions shown in Table S9, section 6 of the Supplementary Appendix) for each model run in order to generate a range of “setting‐scenarios.” These reflect the range of situations in low and low‐middle income settings in southern Africa in terms of epidemic, HIV testing and ART programme characteristics. We generated 1000 such setting‐scenarios in which we track an adult population initially of approximately 20,000 people (with population growth accounted for) and then scaled up the outputs to an adult population the size of Malawi (approximately 10 million). The only constraints beyond those arising from the parameter distributions are that we excluded setting scenarios outside the following ranges: HIV prevalence in 2017 3% to 35%; relative prevalence in 25 to 35 year old women >1.5 times greater than that in 25‐ to 35‐year‐old men; probability of an HIV test when HIV‐related symptoms are present >0.30; number of sex workers who have condomless sex (in our model a sex worker is defined as a woman having had five or more condomless sex partners in any one three‐month period in the past year) 2,500 to 200,000. The characteristics of the 1000 setting‐scenarios in 2017 are presented in Table 1, along with examples of observed data. We consider it plausible that each of the setting‐scenarios represent the real situation in certain sub‐settings in sub‐Saharan Africa defined by geography and demographics.

**Table 1:** Characteristics of the 1000 HIV epidemic/ART programme setting‐scenarios in 2017 (median; 90% range, reflecting variability across setting‐scenarios), in context of country the size of Malawi (adult population age 15 to 65 approx. 10 million)

	Median (90% range) across setting‐scenarios (n = 1000)		Examples of observed data
HIV prevalence (age 15 to 49)	8.2% (4.7% to 17.4%)		Zimbabwe DHS (2015) 14%, Tanzania (2011) 5%, Uganda (2011) 9%, Lesotho (2014)25%^22^.
Relative HIV prevalence by age and sex (relative to men age 25 to 34)	Women	Men	Zimbabwe DHS 2015 men age 15 to 24 0.29, age 35 to 44 2.18, age 45 to 54 2.52; women age 15 to 24 0.69, age 25 to 34 1.82, age 35 to 45 2.88^22^
15 to 24	0.52 (0.28 to 0.77)	0.22 (0.12 to 0.33)	
25 to 34	1.88 (1.56 to 2.38)	1.00	
35 to 44	3.11 (2.37 to 4.41)	2.15 (1.67 to 3.01)	
45 to 54	2.62 (1.85 to 4.16)	1.91 (1.37 to 2.90)	
55 to 64	1.12 (0.69 to 1.88)	1.12 (0.73 to 1.69)	
Prevalence of undiagnosed HIV			
Overall	1.5% (0.7% to 3.5%)		Malawi 2.9%, Zimbabwe 3.8%, Zambia 4.0 (PHIA 2016)^23^ Rwanda ~ 0.3% (Nsanzimana^24^) (Survey estimates could be over‐estimates due to undisclosed diagnosed HIV; Kim et al^25^)
Women	1.2% (0.5% to 2.9%)		
Men	1.9% (0.8% to 4.3%)		
Time since infection among undiagnosed population			
Women			No data known to be available
<1 year	38% (25% to 51%)		
1 to 5 years	41% (32% to 52%)		
≥5 years	20% (11% to 33%)		
Men			
<1 year	24% (15% to 34%)		
1 to 5 years	50% (41% to 57%)		
≥5 years	26% (14% to 40%)		
HIV incidence (age 15 to 49) per 100 person years	0.64 (0.25 to 1.52)		MPHIA (0.37%), ZAMPHIA (0.66%), ZIMPHIA (0.45%)^23^, Swaziland 2.4% (Justman^26^), Mbongolwane and Eshowe, KZN 1.2% (Huerga^27^)
Number of HIV tests in year			
All adults^a^	2,641,000 (1,397,000 to 3,710,000)		Zimbabwe 2.1 m (2016)^28^, Malawi 2.2 m (2015)^29^
Women overall^a^	1,787,000 (1,004,000 to 2,455,000)		
Men overall^a^	835,000 (363,000 to 1,298,000)		
ANC	650,000 (317,000 to 1,115,000)		
FSW	32,000 (9,000 to 93,000)		
Symptomatic	232,000 (221,000 to 247,000)		
Men for VMMC	72,000 (66,000 to 76,000)		
Testing beyond core‐testing^b^			
All adults	1,602,000 (438,000 to 2,652,000)		
Women	961,000 (271,000 to 1,552,000)		
Men	649,000 (165,000 to 1,115,000)		
Percentage of tests resulting in HIV diagnosis			
All adults^a^	2.9% (1.2% to 7.7%)		Estimates are susceptible to bias due to re‐diagnosis of people who do not report previous diagnosis. 6% to 55% depending on group (Sharma et al^5^).
Women overall^a^	2.6% (1.0% to 7.7%)		
Men overall^a^	3.5% (1.4% to 9.1%)		
ANC	2.8% (0.8% to 22.9%)		
FSW	33.7% (10.8% to 49.2%)		
Symptomatic	7.7% (3.3% to 16.6%)		
Men for VMMC	1.3% (0.4% to 3.6%)		
Testing beyond core‐testing^b^			
All adults	1.7% (0.8% to 4.1%)		
Women	1.4% (0.6% to 3.6%)		
Men	2.2% (1.0% to 5.3%)		
Cost of testing per new HIV diagnosis^c^			
All adults^a^	$159 ($73 to $357)		Few estimates reported. Estimates are susceptible to bias described above. >$500 (Bogart 2017; fisher folk Uganda^30^). $36 (Rutstein 2014; partner notification Malawi^31^); $157‐$189 in 2010 (Grabbe; mobile and stand‐alone HIV counselling and testing approaches in Kenya^32^); $25‐$76 (Maheswaran; facility based testing in Malawi^33^).
Women overall^a^	$188 ($81 to $452)		
Men overall^a^	$133 ($63 to $314)		
ANC	$233 ($95 to $650)		
FSW	$30 ($27 to $48)		
Symptomatic	$71 ($42 to $157)		
Men for VMMC	$139 ($112 to $161)		
Testing beyond core‐testing^b^			
All adults	$253 ($115 to $540))		
Women	$336 ($135 to $792)		
Men	$214 ($101 to $472)		
Proportion tested			Zimbabwe DHS 2015 49% women, 36% men (age 15 to 49). Namibia DHS 2013 49% women, 38% men (age 15 to 49). Nigeria DHS 2013 10% women, 10% men^22^
Overall in past year women age 15 to 49	37% (22% to 48%)		
Overall in past year males age 15 to 49	16% (7% to 25%)		
Among those symptomatic with			
HIV symptoms^d^	90% (46% to 93%)		
In pregnancy	92% (89% to 93%)		
FSW (proportion tested in each 3 month period)	25% (17% to 41%)		
Of HIV‐positive people, proportion diagnosed			
Men	73% (59% to 82%)		MPHIA 2016 Malawi (73%; 76% in women, 67% in men), ZAMPHIA 2016 Zambia (67%), ZIMPHIA 2016 Zimbabwe (74%)^23^, Huerga (75%)^27^, Maman (77%)^34^, Gaolathe (78%, higher in women than men)^35^. (Survey estimates likely to be over‐estimates due to undisclosed diagnosed HIV; Kim et al^25^)
Women	89% (82% to 93%)		
Proportion of diagnosed people on ART	83% (66% to 92%)		Zimbabwe 87% (ZIMPHIA), Malawi 89% (MPHIA), Zambia 85% (ZAMPHIA)^23^, Botswana 85% (Gaolathe)^35^.
Proportion of people on ART with VL <1000 cps/mL	88% (84% to 92%)		World Bank South Africa (60% to 88% over districts), ZAMPHIA (89%), MPHIA (91%), ZIMPHIA (87%)^22^, Maman (91%)^34^, Huerga (90%)^27^, Brown (90%)^36^, Botswana 94% (Gaolathe; among citizens of Botswana)^35^

ANC, ante‐natal clinic; FSW, female sex worker; VMMC, voluntary medical male circumcision.

^a^This relates to all testing, not only core‐testing; ^b^testing apart from in FSW, symptomatic, ANC, VMMC; ^c^if cost per test = $3.70; ^d^symptoms of a WHO stage 3 or 4 condition.

For each setting‐scenario, we used the model to make projections for 50 years from 2018 to 2068 under two policies. Under both policies, “core” testing (hereafter referred to as core‐testing) is assumed to be offered; that is testing in pregnant women (one per pregnancy, although many countries aim for two or three tests per pregnancy), for diagnosis of symptoms, every six months in sex workers who have condomless sex (although this is not currently happening in many settings), and in men coming forward for circumcision. In the reference policy, there is no additional HIV testing beyond core‐testing (so in some setting‐scenarios this means less testing than pre‐2018), while in the “additional testing” policy (policy hereafter referred to as additional‐testing) there is additional testing. The characteristics of the additional testing differed for each setting‐scenario; we selected at random a rate of testing per three‐month period and a relative probability of HIV‐positive people being tested compared with HIV‐negative people (which is possible as the true status of a “person” in the model is known). This random selection was from relative rates of testing of 0.1, 0.333, 1, 3, 10 and 30 times the existing testing rate in 2017, and relative probabilities of HIV‐positive people being tested ranging from 1, to 10, 100 and 1000 times the probability of HIV‐negative people being tested. This was done in order to generate across setting‐scenarios various different proportions of tests which result in diagnosis. We chose core‐testing as the reference policy rather than the current testing because we did not want to make any implicit assumption about cost‐effectiveness of current testing beyond core‐testing. We only included setting scenarios in which the number of additional new HIV diagnoses per year is at least 5000. In two‐thirds of the 1000 model runs (setting‐scenarios), we restricted additional‐testing to men or women only, while in the remainder additional‐testing was in both sexes. We assume that the criteria for ART initiation followed those from Malawi up to 2017 and that all people are eligible for ART at diagnosis from 2017 and that viral load monitoring is used from 2017. For each of the setting scenarios we ran 10 replications of each policy to reduce influence of stochastic effects, which was adjudged to be sufficient based on the relative smoothness of the derived relationship between cost‐per‐diagnosis and the median ICER (Table 3).

We describe the effect of the additional‐testing policy on intermediate measures including the mean proportion of people testing in the past year, the proportion of HIV tests resulting in a diagnosis, the cost of HIV testing per new diagnosis (hereafter referred to as cost‐per‐diagnosis), and the percentage of HIV‐positive people diagnosed over the five‐year period from 2018 to 2023. The cost‐per‐new‐diagnosis was based on the assumed testing cost of $3.70 (Clinton Health Access Initiative, personal communication, which is consistent with previous costs in the region at high‐volume facilities^9^), with a total testing cost of $25 for people diagnosed positive, due to the need for confirmatory testing and appropriate counselling. Costs are in 2018 US dollars. A five‐year period was chosen to allow a sufficient time to fully characterize the additional‐testing policy. The testing costs included in the analysis are conceived of as the fully loaded costs of a test being done, including any demand generation, supply chain, fuel, wastage, human resources in administering the test as well as the kit itself. The annual cost of the first‐line regimen of efavirenz, lamivudine, tenofovir is $100 per person per year (with dolutegravir replacing efavirenz by 2019, with a total regimen cost of $75 per person year), programme costs for clinic visits, not including drug, viral load, or CD4 count tests, are $20 per three months^10^, ^11^ with an assumed reduction to $10 per three months when viral load is <1000 copies/mL^8^. Disability weights to calculate DALYs were derived from a comprehensive study^12^.

In order to generate a range of situations with regard to cost per unit test we considered a range of plausible unit costs of HIV tests in addition to the baseline cost of $3.70: $1, $2, $5, $7, $10, $12 and increments of $3 up to $36. The higher unit costs are conceived of as relating to situations in which there are substantial costs in identifying and reaching people to target for testing (e.g. contact tracing). This was applied for all HIV tests, not just the additional tests and includes all costs relating to performing a test, not just the cost for the kit itself. A total of 16,000 setting‐scenario/test unit cost combinations were therefore considered (16 different unit costs for each of 1000 setting‐scenarios). For each of these we calculate the cost‐per‐diagnosis resulting from the additional‐testing as the ratio between the cumulative (undiscounted) cost of additional‐testing and the number of diagnoses due to additional‐testing, averaged over the five years 2018 to 2023. We then assessed the relationship between the cost‐per‐diagnosis and the incremental cost‐effectiveness ratio (ICER) for additional‐testing over core‐testing alone, with the ICER calculated over the 50 year time horizon. The ICER takes account of all costs, including downstream costs (and potential savings in downstream costs) resulting from the testing and diagnosis. Unlike the cost‐per‐diagnosis, the cost‐per‐DALY‐averted cannot be readily measured by programmes and used directly to monitor them.

We take a healthcare perspective for our cost‐effectiveness analysis. The health‐financing environment for HIV in Malawi and other countries is complex, with international funding initiatives channelled particularly to support HIV testing. In each setting‐scenario/unit test cost combination, additional‐testing was deemed to be cost‐effective if the ICER was below $500 per DALY averted or if there was a saving in cost with the additional‐testing policy and DALYs were averted. This use of the cost‐effectiveness threshold reflects the health foregone (opportunity costs) due to resources committed to HIV testing consequentially being unavailable to provide other HIV interventions (so that $500 reflects the cost‐per‐DALY‐averted of these foregone activities)^13^, ^14^. Severe constraints on overall healthcare spending in low‐income countries in the region, and Malawi^15^ in particular, mean that this cost‐effectiveness threshold is only likely to be relevant for resource allocation within the HIV programme which is overwhelmingly reliant on overseas aid^16^. The Ministry of Health in Malawi also has to support the delivery of other (non‐HIV) healthcare interventions that generate health gains at less than this amount remain, for example diagnosis and treatment for hypertension. The cost‐effectiveness threshold reflecting the resource allocation decisions it faces is therefore likely to be much lower. This implies more health could be gained using some HIV funding for other pressing healthcare needs. Where delivery of HIV interventions draws upon resources that would otherwise be used for other health activities such as the use of staff resources a lower threshold would probably be required, and we use $300 and $150 in sensitivity analyses. We follow convention and use a discount rate of 3% per annum for both costs and outcomes in the main analysis^17^, although real rates of interest faced by governments in the region are often much higher than this. Therefore, we also consider discount rates of 0% and 10% in sensitivity analyses. All costs are in U. S dollars.

We fitted a logistic curve for the relationship over setting‐scenarios between the cost‐per‐diagnosis and whether the ICER was below $500 or not (the binary outcome variable), in order to derive the median (and 80% centile) cost of testing per new HIV diagnosis across setting scenario/unit test cost combinations.

Finally, since our core‐testing comparison includes six monthly testing in sex workers, we repeated the above‐described process but instead of comparing core‐testing‐only with core‐testing‐plus‐additional‐testing we compared core‐testing with core‐testing minus targeted‐testing in sex workers. This was to assess our assumption that testing in sex workers is cost‐effective.

Ethical approval was not relevant for this study as it does not involve human subjects.

## 3 Results

The characteristics of the 1000 setting‐scenarios in 2017, before the introduction of our specified testing policies of core‐testing and core‐testing plus additional‐testing, are presented in Table 1. Table 2 shows the undiscounted median and 90% range over setting‐scenarios of the effect of the additional‐testing policy on a range of outputs over the first five years from initiation of the policy (i.e. 2018 to 2023). For women, results are based only on setting‐scenarios in which there is additional‐testing in women, irrespective of whether or not there is also additional‐testing in men. The same applies to men. The number of additional tests over and above core‐testing of 496,000 (27,000 to 4,211,000) in women and 371,000 (25,000 to 4,295,000) in men represents an 8% (90% range +0.4% to 67%) higher proportion of women tested per year, and 6% (90% range +0.4% to 75%) higher proportion of men tested per year respectively. With the policy of additional‐testing, the proportion of people with HIV who are diagnosed (average 2018 to 2023) is a median of 7% higher in women and 21% in men. The median cost‐per‐diagnosis of additional‐testing was $399 (90% range $25 to $7,187) in women and $288 ($21 to $4,975) in men. We also calculated the undiscounted cost‐per‐diagnosis over the full 50 years from 2018 to 2068 and the median cost was 1.24 times greater than the cost over the first five years.

**Table 2:** Effect of additional‐testing on number of tests, testing rates and proportion diagnosed 2018 to 2023 (median & 90% range across 1000 setting‐scenarios of the mean value over 2018 to 2023, reflecting variability across setting scenarios)

	Effect of additional‐testing^a^
Number of HIV tests/year	
Women^b^	+496,000 (+27,000 to +4,211,000)
Men^c^	+371,000 (+25,000 to +4,295,000)
Number of new diagnoses per year	
Women^b^	+20,370 (+7,200 to +49,470)
Men^c^	+28,890 (+8,980 to +67,210)
Proportion tested in the past year (age 15 to 49)	
Women^b^	+8% (+0.4% to +67%)
Men^c^	+6% (+0.4% to +75%)
Of HIV‐positive people, proportion diagnosed^d^	
Women^b^	+7% (+2% to +12%)
Men^c^	+21% (+5% to +33%)
Cost‐per‐diagnosis with additional‐testing^e^	
Women^b^	$399 ($25 to $7,187)
Men^c^	$288 ($21 to $4,975)

^a^Within‐scenario (model run) difference; ^b^across 667 setting‐scenarios in which additional‐testing is done in women and men or in women only; ^c^across 667 setting‐scenarios in which additional‐testing is done in women and men or in men only; ^d^The effect of additional‐testing is for the proportion of setting scenarios in which the proportion diagnosed is >90% to increase from 0% to 65% in men, and from 29% to 94% in women; ^e^over 10,672 (=667 × 16) setting‐scenario/test unit cost combinations.

**Figure 1:**
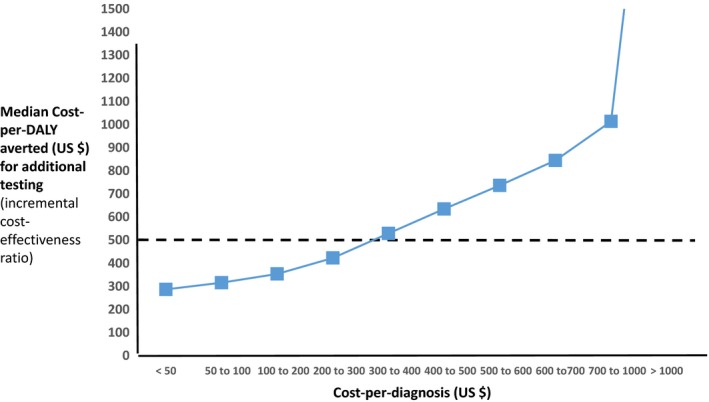
Relationship between cost‐per‐diagnosis and cost‐per‐DALY averted for additional‐testing Over 16,000 setting‐scenario – test unit cost combinations.

**Figure 2:**
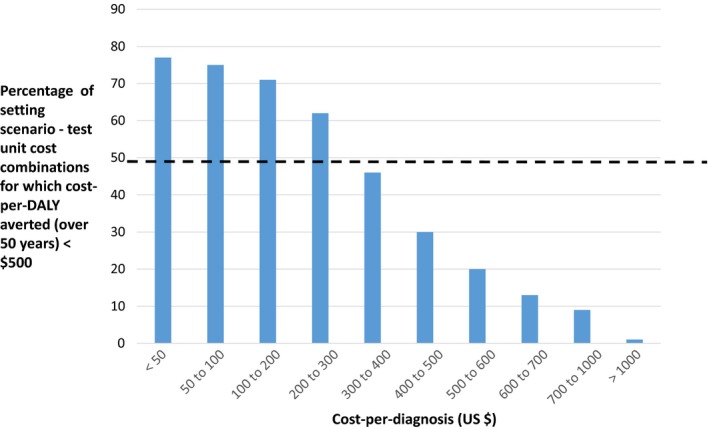
Relationship between cost‐per‐diagnosis and cost‐effectiveness of additional‐testing Over 16,000 setting‐scenario – test unit cost combinations.

In Table 3 and Figure 1, we present the median ICER (cost‐per‐DALY averted) across setting‐scenario/test unit cost combinations according to the cost‐per‐diagnosis. We also show the proportion of such combinations in which additional‐testing is cost‐effective, based on a cost‐effectiveness threshold of $500 (also shown in Figure 2). Considering additional‐testing overall (additional‐testing in both men and women, in women alone, or in men alone), there is a strong graded relationship between the cost‐per‐diagnosis and the ICER. In around 50% of combinations additional‐testing is cost effective when the cost‐per‐diagnosis is $300 to $400 per new diagnosis ($315 based on fitting a logistic curve; 80% centile $17).

**Table 3:** Cost‐effectiveness of *additional‐testing* beyond core testing and median incremental cost effectiveness ratio (ICER; cost‐per‐DALY averted) according to Cost‐per‐diagnosis

Cost‐per‐diagnosis (2018 to 2023)^a^	Percent of setting‐scenarios in which additional‐testing is cost effective^b^	Median ICER
(a) overall (additional‐testing in both men and women, in women alone, or in men alone)		
<$50	77% (1701/2203)	$287
$50 to $100	75% (1062/1411)	$316
$100 to $200	71% (1612/2283)	$354
$200 to $300	62% (1159/1872)	$423
$300 to $400	46% (481/1053)	$529
$400 to $500	30% (218/734)	$635
$500 to $600	20% (125/618)	$737
$600 to $700	13% (60/454)	$845
$700 to $1000	9% (81/937)	$1014
>$1000	1% (35/4435)	$3069
(b) additional‐testing in women only		
<$50	43% (321/741)	$541
$50 to $100	35% (163/454)	$602
$100 to $200	33% (246/748)	$661
$200 to $300	25% (154/619)	$751
$300 to $400	10% (32/333)	$1049
$400 to $500	5% (13/238)	$1173
$500 to $600	5% (10/204)	$1282
$600 to $700	3% (5/148)	$1403
$700 to $1000	1% (2/311)	$1710
>$1000 to $1500	0% (0/1532)	$4871
(c) additional‐testing in men only		
<$50	94% (742/791)	$189
$50 to $100	95% (462/487)	$215
$100 to $200	91% (743/814)	$243
$200 to $300	89% (581/652)	$288
$300 to $400	78% (260/333)	$372
$400 to $500	58% (135/232)	$463
$500 to $600	44% (84/193)	$542
$600 to $700	29% (40/140)	$623
$700 to $1000	21% (66/308)	$704
>$1000 to $1500	2% (34/1378)	$1914

Considering 16,000 setting‐scenario/test unit cost combinations (1000 setting‐scenarios × 16 different unit costs for testing), 5344 (334 × 16) in which additional‐testing in both men and women is introduced; 5328 (333 × 16) each in which it is only introduced in women; 5328 (333 × 16) each in which it is only introduced in men.

^a^Not discounted; ^b^using $500/DALY cost effectiveness threshold (This is to assess for each setting scenario test cost combination whether additional testing is cost effective).

**Figure 3:**
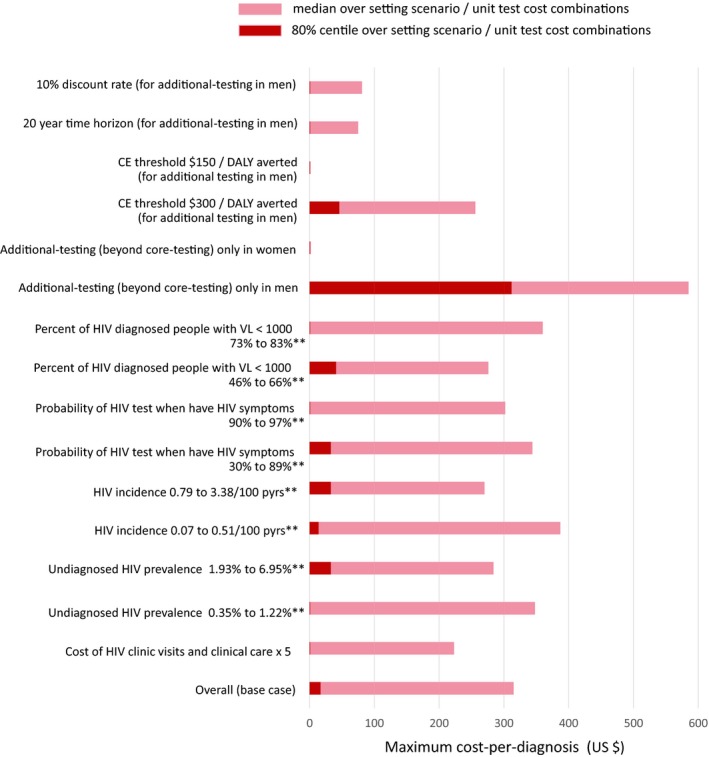
Maximum cost‐per‐diagnosis for testing beyond key groups to be cost‐effective. Variations in sensitivity analysis. *Lower/ **upper tertile of the distribution across setting scenarios in 2017. No bar in red indicates that for over 20% of setting scenario / unit test cost combinations there is no cost of testing per diagnosis at which testing is cost‐effective.

Figure 3 shows the median (80% centile) across setting scenario/unit test cost combinations for the maximum cost‐per‐diagnosis that allowed additional‐testing to still be cost‐effective according to various factors. Importantly, this threshold cost‐per‐diagnosis did not vary substantially according to several epidemic and programmatic features in 2017, namely the proportion of diagnosed people with viral suppression (a measure of the overall quality of on ART programme), proportion of people with symptoms due to HIV who are tested as a result, the prevalence of undiagnosed HIV, and HIV incidence in 2017. It also does not vary substantially according to the test unit cost. This result suggests that the relationship we describe is likely to hold across the diverse settings in southern Africa shown in Table 1.

When we restricted additional‐testing to women only, there remained a strong relationship between the cost‐per‐diagnosis and the ICER, but the median ICER was above the $500 threshold even in the lowest category of cost‐per‐diagnosis and the proportion of setting‐scenario/test‐cost combinations in which additional‐testing was cost‐effective was always below 50%. This implies that additional‐testing was not cost‐effective regardless of the cost‐per‐diagnosis (Table 3(b) and Figure 3). In contrast, additional‐testing in men only was cost‐effective up to a cost‐per‐diagnosis of $585 (median; 80% percentile $312). Again, there remained a strong relationship between the cost per new diagnosis and the ICER.

The maximum cost‐per‐diagnosis for additional‐testing to remain cost‐effective fell to $256 (median; 80% centile $46) when the cost‐effectiveness threshold was set to $300 per DALY averted; with a cost‐effectiveness threshold of $150, additional‐testing in men was not cost‐effective. When considering a discount rate of 10% per annum or a shorter time horizon of 20 years for testing, the maximum cost‐per‐diagnosis for additional‐testing in men to remain cost effective was below $100 (median). In sensitivity analyses in which we used a 0% discount rate instead of 3%, the median for the maximum cost‐per‐diagnosis for testing in men was $821.

Lastly, in our comparison of core‐testing with core‐testing‐minus‐targeted‐testing‐in‐sex workers, we found mean incremental cost‐effectiveness ratio which ranged from $115 per DALY averted, if the unit cost of testing is $1, to $249, if the unit cost of testing is $36, suggesting that six monthly testing in sex workers is indeed cost‐effective.

## 4 Discussion

Countries require appropriate metrics to assess their HIV testing programmes that can be measured. Our analysis confirms that the cost‐per‐diagnosis is a key metric that is strongly predictive of the overall cost‐effectiveness of an HIV testing programme. We found that HIV testing programmes increasing testing in men in low‐income settings in southern Africa are on average cost‐effective if they cost below $585 per new diagnosis. If testing due to symptoms, in pregnancy and in female sex workers having condomless sex (100% testing every six months) is in place, testing women as part of further population testing programmes is generally unlikely to be cost‐effective. In reality, programmes providing regular testing for sex workers are not as widespread as recommended. If such a testing programme in sex workers is not in place then our results indicate such programmes should be introduced. Our estimates for the cost‐per‐diagnosis did not vary substantially according to epidemiologic and programmatic characteristics and so appear to be generalizable to lower income countries in southern Africa, and likely elsewhere in sub‐Saharan Africa. This is a measure that is increasingly being reported by studies^9^, ^18^, ^19^, ^20^, ^21^.

In addition to $500, we also considered a cost‐effectiveness threshold of $300, which recognizes that HIV programmes can draw resources from other healthcare areas. A threshold of $300 is still high from an overall perspective of healthcare provision in Malawi and this suggests that new HIV diagnoses in men need to be achieved at an average cost below $256 (median over setting scenario/unit test cost combinations). With a cost‐effectiveness threshold of $150, which is closer to that which applies in Malawi for overall healthcare provision^15^, additional‐testing was not cost‐effective. Since we present the relationship between the cost‐per‐diagnosis and the ICER, country programmes can apply other cost‐effectiveness thresholds and may have evidence on opportunity costs in their particular setting. We calculated the cost‐per‐diagnosis over the first five years (2018 to 2023) but this can change over the subsequent 45 years of the total projection; over the full 50 years this was a median of 1.24 times greater than the cost over the first five years. For this reason it is conceivable that short‐term testing programmes (e.g. lasting 5 years and stopping in 2023) in men that cost somewhat more than $585 per newly diagnosed man may nevertheless be cost‐effective.

Implementation of our results requires that, besides being able to estimate the full cost of testing, programmes can also reliably estimate the number of new HIV diagnoses in a given period (i.e. without inclusion of people who are being re‐diagnosed having earlier been tested positive). Testing programmes rely on self‐report to separate positive tests done in people who have previously received an HIV diagnosis from those done in people who have never been diagnosed. In some cases an individual may not wish to disclose a previous positive test, which makes this information challenging to collect reliably. When estimating the number of new diagnoses that a programme makes, adjustment will have to be made to account for the fact that a proportion of apparent new diagnoses are in fact not, otherwise cost‐effectiveness will be over‐estimated. That adjustment factor might be informed by data on the ratio of the increase over a period of time in the number of people on ART, compared with the number of new positive tests in that period. This said, the effect of such over ascertainment of new diagnoses could be less important if this diagnosis is the one that leads the person to link to care and start ART. Likewise, if testing programmes can demonstrate linkage of people testing negative to effective prevention this will likely enhance cost‐effectiveness. We have not explicitly considered that additional‐testing may be based on distributing self tests. In the analyses we assume that 5% of people will not agree to test unless they are symptomatic. If self testing is more effective at being able to reach such individuals than conventional testing, or if there are settings were the size of this group is greater than 5%, self testing may have some benefits that are currently not fully captured by this analysis.

Our work has other limitations. Modelling is inevitably an imperfect representation of reality, although we have attempted to convey uncertainty. Future changes in ART programmes are taken into account in so far as we allow the HIV epidemic to play out with people being put onto treatment, interrupting treatment at various possible rates, experiencing resistance mutations so that virologic failure occurs, switching to second line regimens at a range of rates, etc. However, we cannot know which other changes might occur over the coming 50 years. As significant changes from the assumptions occur, this analysis would need updating. In addition, we sampled from an array of parameter distributions and then applied some filtering in order to generate a range of setting‐scenarios which appear to broadly represent the distribution of a number of characteristics.

## 5 Conclusions

For testing programmes in low‐income settings in southern Africa that implement universal eligibility for ART there is an extremely strong relationship between the cost‐per‐diagnosis and the cost‐per‐DALYs averted (ICER), indicating that that the cost‐per‐diagnosis can be used to monitor the cost‐effectiveness of testing programmes.

## Competing interest

No conflicts to declare.

## Authors’ contributions

All authors provided input for analysis, data modelling and for the entire manuscript, and were involved in review and approval of the final version. IJ, TA, CK, JvO, ND, FC, OK, DF, KH,RB, HA, LN, CJ, RB, AF and AJ had direct experience of country testing programmes. AP, VC, FN and LB‐M were involved in development of the underlying transmission model. AP was involved in running of the model.

## Supporting information


**Table S1.** Distribution of ages of simulated individuals in 1989
**Table S2.** Age specific death rates (per year)
**Table S3.** Values of *f*_gij_ (values determining probability of transitioning between short‐term partner risk behaviour groups)
**Table S4.** Values of *r*_ga_ (factor determining relative level of sexual risk activity)
**Table S5.** Percent of newly formed long term partnerships classified into each of three duration groups, each of which has a different tendency to endure (higher class, more durable)
**Table S6.** Sexual mixing by age and gender. The proportion of short term partnerships formed by men in age group a_m_ which are with females of age group a_f_ and the proportion of short‐term partnerships formed by females in age group a_f_ which are with men of age group a_m_
**Table S7.** Rate of WHO stage 4 disease according to CD4 count and viral load
**Table S8.** Example model outputs of incubation period by age.
**Table S9.** Prior distributions for parameters.
**Table S10.** Unit Costs and disability weights for DALYs
**Table S11.** Disability weights
**Figure S1.** Summary of modelling of sexual behaviour and HIV acquisition.
**Figure S2.** Overview of modelling of natural history of HIV infection.
