# Supplementary material for: Cost‐per‐diagnosis as a metric for monitoring cost‐effectiveness of HIV testing programmes in low‐income settings in southern Africa: health economic and modelling analysis
Source: J Int AIDS Soc. 2019 Jul 9;22(7):e25325. doi: 10.1002/jia2.25325 (PMC6615491; doi:10.1002/jia2.25325)
Supplement: Supplementary file 1 — Table S1. Distribution of ages of simulated individuals in 1989 Table S2. Age specific death rates (per year) Table S3. Values of f gij (values determining probability of transitioning between short‐term partner risk behaviour groups) Table S4. Values of r ga (factor determining relative level of sexual risk activity) Table S5. Percent of newly formed long term partnerships classified into each of three duration groups, each of which has a different tendency to endure (higher class, more durable) Table S6. Sexual mixing by age and gender. The proportion of short term partnerships formed by men in age group am which are with females of age group af and the proportion of short‐term partnerships formed by females in age group af which are with men of age group am Table S7. Rate of WHO stage 4 disease according to CD4 count and viral load Table S8. Example model outputs of incubation period by age. Table S9. Prior distributions for parameters. Table S10. Unit Costs and disability weights for DALYs Table S11. Disability weights Figure S1. Summary of modelling of sexual behaviour and HIV acquisition. Figure S2. Overview of modelling of natural history of HIV infection. [file JIA2-22-e25325-s001.docx]

Cost- per-diagnosis as a metric for monitoring cost effectiveness of testing programmes in low income settings in southern Africa: health economic and modelling analysis

Supporting Information

**1. Introduction to the approach taken**

The HIV Synthesis Transmission model is an individual-based stochastic model of heterosexual transmission, progression and treatment of HIV infection within a southern African context which has been used previously to consider a range of questions in this region (e.g. Cambiano et al 2013, 2014, 2015, Phillips et al 2008, 2011, 2014, 2018). Details of the model are given in subsequent sections below. For this project we based the demographics of the population studied and HIV epidemic features around those for Malawi and the calibration to data is shown above. However, in addition, by sampling widely from the parameter distributions we also generated diverse setting scenarios in respect of many epidemic aspects, such as sexual behaviour, HIV prevalence, ART uptake and HIV incidence.

**2. Demographic model**

**General population death rates and determination of age in 1989**

The model runs to from 1989 (the start of the epidemic) to 2039 (although in our results we concentrate on the period to 2030), with variables updated in 3 month periods. Each run of the simulation program creates 100,000 simulated people who will be age 15 or above at some point between 1989 and 2039, of whom approximately 35,000 are alive and age over 15 at any one point in time. In order to scale up from the simulated population to a population size of 10 million we use a scale factor of 267.

The initial age distribution for both males and females is determined on the basis of the distribution in Table S1.

**Table S1.** Distribution of ages of simulated individuals in 1989

Probability

Age group of being in age

group in 1989

-------------------------------------------

-35-14 0.770

15-24 0.083

25-34 0.065

35-44 0.041

45-54 0.025

55-64 0.015

--------------------------------------------

This distribution is chosen such that in the absence of HIV, given the death rates below, the population size increases over time, as is projected in Malawi and, to some extent, other countries in the region. Thus around 77% of simulated people have an age below 15 in 1989 (and most are yet to be born). The only variable that is modelled and updated up to reaching the age of 15 (when becoming potentially sexually active) is age itself. The “youngest” person in 1989 is age -35 (i.e. will be born in 2024 and reach age 15 in 2039, when the modelled period ends.

Age specific death rates for uninfected people are based on death rates in South Africa in 1997 (Table S2) – before the significant impact of HIV-related deaths. For the context of Malawi, these death rate are multiplied by 2 based on fitting to the population pyramid

<https://www.cia.gov/library/publications/resources/the-world-factbook/geos/mi.html> (people and society).

**Table S2.** Age specific death rates (per year)

Age group Annual death rate

--------------------------------------------

Males

15-19 0.00200

20-24 0.00320

25-29 0.00580

30-34 0.00750

35-39 0.00800

40-44 0.01000

45-49 0.01200

50-54 0.01900

55-59 0.02500

60-64 0.03500

65-69 0.04500

70-74 0.05500

75-79 0.06500

80-84 0.10000

>85 0.40000

Females

15-19 0.00150

20-24 0.00280

25-29 0.00400

30-34 0.00400

35-39 0.00420

40-44 0.00550

45-49 0.00750

50-54 0.01100

55-59 0.02000

60-64 0.02100

65-69 0.04000

70-74 0.03800

75-79 0.05000

80-84 0.07000

>85 0.15000

--------------------------------------------

**3. Sexual behaviour and risk of HIV acquisition**

Here we describe the approach to modelling sexual behaviour and HIV acquisition. The basic approach is summarized in Figure S1. Sexual behaviour is characterized by two variables representing, respectively, the number of short term condomless sex partners and whether the person has a current long term condomless *sex* partners in the 3 month period. The status of long term partners is tracked over time (i.e. if they are infected, diagnosed, on ART). Short term partners are not tracked over time, in that if a person has a short term partner in time period t who is infected with HIV, this is independent of the probability that any short term partner in time t+1 is infected with HIV. Many of the parameters described below were sampled from distributions for each run of the model in order to generate an array of different scenarios, as described below.

**
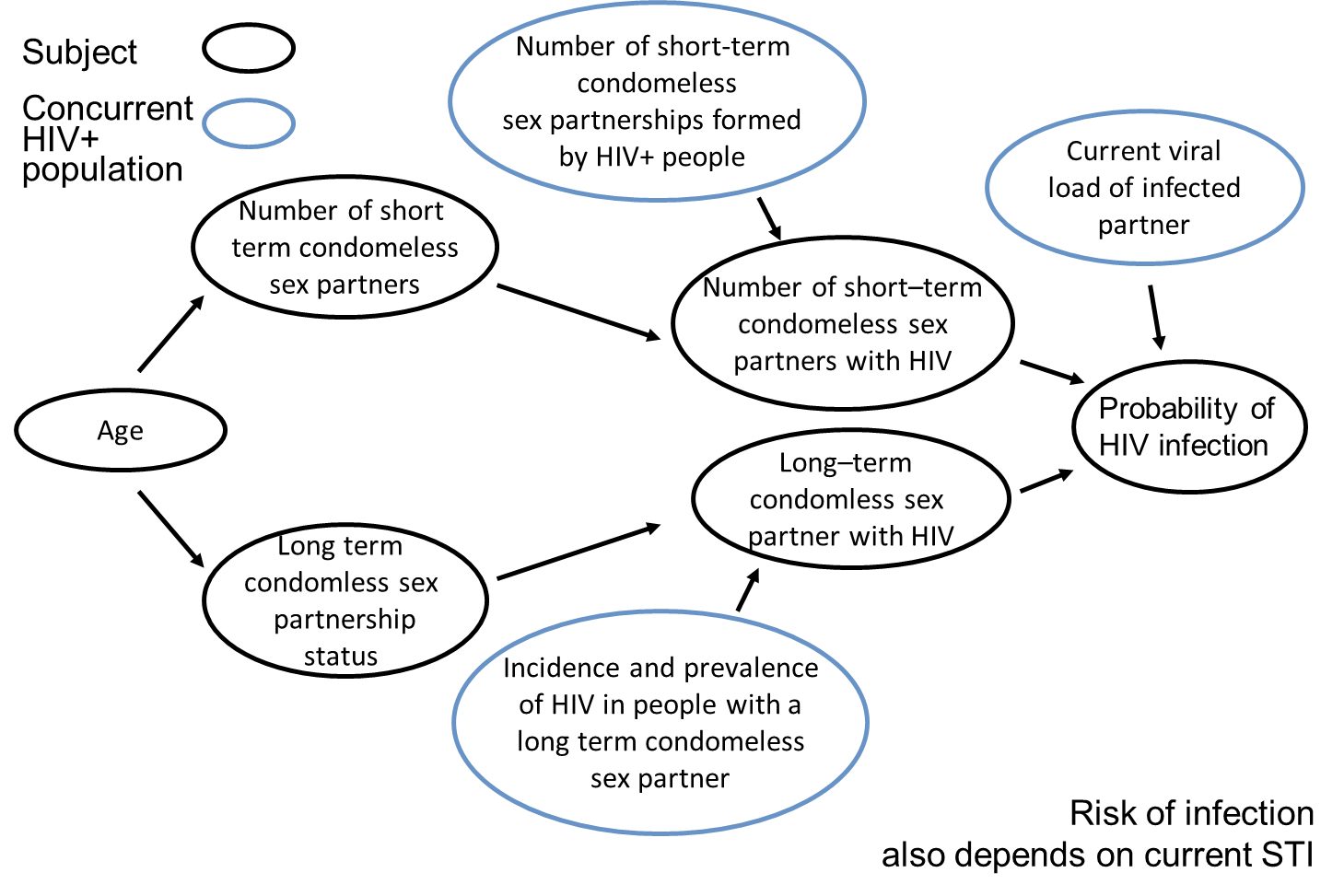
Figure S1.** Summary of modelling of sexual behaviour and HIV acquisition

**Determination of number of short term (condomless sex) partners at period t**

Numbers of short term partners in a given period was generated at random, according to which of four sexual behaviour groups the person was in for this period. Changes in the sexual behaviour group from t-1 to t were determined by transition probabilities between 4 groups: (i) no short term condomless partners in 3 month period, (ii) 1 short term partner, (iii) medium number of short term partners, and (iv) high number of short term partners. Transition probabilities $p_{gija}$ of moving from partner group i at t-1 to partner group j at t are given by

$p_{\mathrm{gija}}=\frac{f_{\mathrm{gij}}}{\left( f_{gi1}+\sum_{j=2}^{4} \left( f_{\mathrm{gij}}\cdot r_{\mathrm{ga}} \right) \right)} \text{ for }j=1$ $p_{gija}=\frac{f_{gij}\times r_{ga}}{\left( f_{gi1}+\sum_{j=2}^{4} \left( f_{gij}\cdot r_{ga} \right) \right)}$

where g = 0,1 for males, females, respectively, and a = 1-10 for age groups 15-, 20-, 25-, 30-, 35-, 40-, 45-, 50-, 55-, 60-, respectively. Values of $f_{gij}$ and $r_{ga}$ are given in Tables S3 and S4, respectively, and if j=1 then $r_{ga}$=1.

We considered nine sets of values of $f_{gij}$ each for males and females as shown in Table S3, characterized by substantially different intra-person variability over time in sexual behaviour subgroups, and sampled a matrix at random independently for each gender for each model run.

Values of $r_{ga}$ are modified at time t by a factor 0.2 if the subject has a current AIDS defining disease and by a factor *ch_risk_diag_newp* (with a value 0.83, informed by (Fonner et al 2012) if the subject is diagnosed with HIV (sqrt(*ch_risk_diag_newp* from 6 months after diagnosis). In addition, there is a person-fixed modification factor. For a proportion *p_rred_p* of men and 1.5.*p_rred_p* of women, values of $r_{ga}$ are modified by a factor 0.1, to reflect the fact that a proportion of people experience only very low sexual risk activity in their life. Similarly for a proportion *p_hsb_p* values of $r_{ga}$ are modified by a factor 3 in women, representing women with a higher chance of becoming sex workers. The value of these parameters is sampled from a distribution at the start of each model run (see below).

Actual transitions between groups were determined by random sampling. For the first two groups the number of partners in the period is given (i.e. no short term partners, 1 short term partner, respectively). When a person was in the medium short term partners group the number of partners was determined by sampling from a Poisson(*highsa*), where *highsa*. When in the high short term partners group the number of partners was determined by sampling from a Poisson(2) distribution and multiplied by the parameter *swn*. The value of these parameters is also sampled from a distribution at the start of each model run (see below).

**Table S3.** Values of $f_{gij}$ (values determining probability of transitioning between short term partner risk behaviour groups)

Sexual behaviour transition matrices for **females**

--------------------------------------------------------------------------------------------------------------------

Short term partners Short term partners group in period t

group in period t-1 0 1 medium high

(Poisson (Poisson

mean *highsa)* mean 2 x *swn)*

--------------------------------------------------------------------------------------------------------------------

Sexual behaviour transition matrix 1

0 0.99 0.005 0.005 0.0001

1 0.95 0.04 0.01 0.0001

medium 0.025 0.03 0.95 0.0005

high 0.01 0.01 0.03 0.95

Sexual behaviour transition matrix 2

0 0.98 0.01 0.01 0.0005

1 0.95 0.04 0.01 0.0005

medium 0.03 0.07 0.90 0.0025

high 0.02 0.03 0.05 0.90

Sexual behaviour transition matrix 3

0 0.95 0.025 0.025 0.001

1 0.90 0.07 0.03 0.001

medium 0.10 0.10 0.80 0.005

high 0.06 0.06 0.08 0.80

Sexual behaviour transition matrix 4

0 0.99 0.005 0.005 0.0002

1 0.95 0.04 0.01 0.0002

medium 0.025 0.02 0.95 0.001

high 0.01 0.01 0.03 0.95

Sexual behaviour transition matrix 5

0 0.98 0.01 0.01 0.001

1 0.95 0.04 0.01 0.001

medium 0.03 0.07 0.90 0.005

high 0.02 0.03 0.05 0.900

Sexual behaviour transition matrix 6

0 0.95 0.025 0.025 0.002

1 0.90 0.07 0.03 0.002

medium 0.10 0.10 0.80 0.01

high 0.06 0.06 0.08 0.80

Sexual behaviour transition matrix 7

0 0.99 0.005 0.005 0.00005

1 0.95 0.04 0.01 0.00005

medium 0.025 0.02 0.95 0.00025

high 0.01 0.01 0.03 0.95

Sexual behaviour transition matrix 8

0 0.98 0.01 0.01 0.00025

1 0.95 0.04 0.01 0.00025

medium 0.03 0.07 0.90 0.00125

high 0.02 0.03 0.05 0.90

Sexual behaviour transition matrix 9

0 0.95 0.025 0.025 0.0005

1 0.90 0.07 0.03 0.0005

medium 0.10 0.10 0.80 0.0025

high 0.06 0.06 0.08 0.800

-----------------------------------------------------------------------------------------------------------------------

Sexual behaviour transition matrices for **males**

--------------------------------------------------------------------------------------------------------------------

Short term partners Short term partners group in period t

group in period t-1 0 1 medium high

(Poisson (Poisson

mean *highsa)* mean 2 x *swn)*

--------------------------------------------------------------------------------------------------------------------

Sexual behaviour transition matrix 1

0 0.99 0.005 0.005 0.00005

1 0.88 0.10 0.02 0.00005

medium 0.03 0.07 0.90 0.00025

high 0.00 0.00 0.00 1.00000

Sexual behaviour transition matrix 2

0 0.98 0.01 0.01 0.00025

1 0.90 0.08 0.02 0.00025

medium 0.05 0.15 0.80 0.000125

high 0.00 0.00 0.00 1.00000

Sexual behaviour transition matrix 3

0 0.95 0.03 0.02 0.0005

1 0.93 0.05 0.02 0.0005

medium 0.20 0.20 0.60 0.0025

high 0.00 0.00 0.00 1.0000

Sexual behaviour transition matrix 4

0 0.99 0.005 0.005 0.0001

1 0.88 0.10 0.02 0.0001

medium 0.03 0.07 0.90 0.0005

high 0.04 0.04 0.09 0.83

Sexual behaviour transition matrix 5

0 0.98 0.01 0.01 0.005

1 0.90 0.08 0.02 0.0005

medium 0.05 0.15 0.80 0.0025

high 0.025 0.06 0.17 0.75

Sexual behaviour transition matrix 6

0 0.95 0.03 0.02 0.001

1 0.93 0.05 0.02 0.001

medium 0.20 0.20 0.60 0.005

high 0.04 0.08 0.21 0.67

Sexual behaviour transition matrix 7

0 0.99 0.005 0.005 0.000025

1 0.88 0.10 0.02 0.000025

medium 0.03 0.07 0.90 0.000125

high 0.00 0.00 0.00 1.00000

Sexual behaviour transition matrix 8

0 0.98 0.01 0.01 0.000125

1 0.90 0.08 0.02 0.000125

medium 0.05 0.15 0.80 0.000625

high 0.00 0.00 0.00 1.00000

Sexual behaviour transition matrix 9

0 0.95 0.03 0.02 0.00025

1 0.93 0.05 0.02 0.00025

medium 0.20 0.20 0.60 0.00125

high 0.00 0.00 0.00 1.00000

-----------------------------------------------------------------------------------------------------------------------

**Table S4.** Values of $r_{ga}$ (factor determining relative level of sexual risk activity)

------------------------------------------------------------------------------------------

Age group Males females

(a=1,10) (g=1) (g=2)

------------------------------------------------------------------------------------------

15- 0.60 1.60

20- 0.60 1.60

25- 1.00 1.00

30- 0.80 0.80

35- 0.65 0.50

40- 0.50 0.35

45- 0.40 0.10

50- 0.35 0.05

55- 0.25 0.04

60- 0.15 0.02

------------------------------------------------------------------------------------------

**Determination of having a long term (condomless sex) partner at period t**

Note that only condomless sex partnerships are modelled. Thus if a person has a long term partner but condoms are used on all occasions of sexual intercourse then this is not counted as having a long term condomless sex partner.

At each period, people with no current long term partner have age-dependent probabilities of having a new long term partner is dependent on parameter *eprate* and given by: age 15-24, p= *eprate*; age 25-34, p= *eprate*; age 35-44, p= *eprate/2*; age 45-54, p= *eprate/3*; age 55-64, p= *eprate/5* (*eprate* = 0.1).

At the time a long term partnership is started, it is classified into 3 duration groups, each with a different tendency to endure. The percent of people in each group is dependent on age and is shown in Table S5.

At time period, t, for people with a long term partner, the probability of the condomless sex partnership continuing is (1-(0.25 / *ch_risk_beh_ep*)) if duration category is 1, is (1-(0.05 / *ch_risk_beh_ep*)) if duration category is 2, and (1-(0.02 / *ch_risk_beh_ep*)) if duration category is 3, where *ch_risk_beh_ep* is a parameter conveying the population level change in sexual behaviour with long term partners that occurs in 1995*.* Further, this probability is reduced by a factor *ch_risk_diag* in the 3 month period after a partner’s diagnosis, if a partner has HIV and is diagnosed.

Note also that levels of sexual behaviour, in terms of numbers of short term partners and the probability of a long term partner are essentially determined by the levels of such sexual behaviour required in order to produce an epidemic as described, given rates of transmission with condomless sex partners. Sexual behaviour tends to be under-reported particularly in women and higher levels of behaviour have to be assumed both to be consistent with levels of risk behaviour reported in men, and to generate an epidemic of the proportions observed (e.g. Gregson 2002, Johnson 2009).

**Table S5.** Percent of newly formed long term partnerships classified into each of three duration groups, each of which has a different tendency to endure (higher class, more durable).

Age 1 2 3

-----------------------------------------------------------------------------------------------

15-44 30% 30% 40%

45-54 30% 50% 20%

55-64 30% 70% 0%

-----------------------------------------------------------------------------------------------

**Population level change in sexual behaviour**

There is assumed to be a general average reduction in condomless sex after 1995, reflecting the reductions observed over the period from around this date (Gregson 2010, Halperin 2011).

**Determination of number of short term (condomless sex) partners who are HIV infected at time t**

For each short term partner that a subject has at time t, the probability that the partner is infected is calculated. This is dependent on the prevalence of HIV in those of the opposite gender, taking consideration of age mixing. If the subject is of gender g and age group a, then for each short term partner the first step is to determine by sampling at random, the age group of the short term partner, $a^{\text{newp }}$(in fact, for simplicity, all short term partners at time t are assumed to be in this same age group). The gender and age mixing probabilities used are given by values in Table S6.

**Table S6**. Sexual mixing by age and gender. The proportion of short term partnerships formed by men in age group a_m_ which are with females of age group a_f_ and the proportion of short term partnerships formed by females in age group a_f_ which are with men of age group a_m_.

Females

Age group (a_f_)

Males

Age group (a_m_) 15-24 25-34 35-44 45-54 55-64

----------------------------------------------------------------------------------------

15-24 0.865 0.11 0.025 0.00 0.00

25-34 0.47 0.43 0.10 0.00 0.00

35-44 0.30 0.50 0.20 0.00 0.00

45-54 0.43 0.30 0.23 0.03 0.01

55-64 0.18 0.18 0.27 0.27 0.10

----------------------------------------------------------------------------------------

Males

Age group (a_m_)

Females

Age group (a_f_) 15-24 25-34 35-44 45-54 55-64

----------------------------------------------------------------------------------------

15-24 0.43 0.34 0.12 0.10 0.01

25-34 0.09 0.49 0.30 0.10 0.02

35-44 0.03 0.25 0.34 0.25 0.13

45-54 0.00 0.00 0.05 0.25 0.70

55-64 0.00 0.00 0.00 0.10 0.90

----------------------------------------------------------------------------------------

Then, for the given partner (of gender 1-g and age group *a^newp^*), the risk that the partner is infected is then given by

$$h_{gat}=\frac{\sum_{a^{\text{newp}},(g-1)} L_{(t-1)}^{\text{inf}}}{\sum_{a^{\text{newp}},(g-1)} L_{(t-1)}}$$

where $L_{(t-1)}^{\text{inf}}$ is the total number of infected short term partners at time (t-1), and $L_{(t-1)}$ is the total number of short term partners at time t-1. The numerator is therefore the total number of infected short term partnerships of the opposite gender in age group *a^newp^_._*

Since we assume that all short term partners at time t are in this same age group, the total number of infected short term partners that the subject has at time t, $L_{t}^{\text{inf}}$, is then given by

$$L_{t}^{\text{inf}}=\text{Min}\left( \text{Poisson}\left( h_{t}\cdot L_{t} \right),L_{t} \right)$$

**Determination of probability that a long term partner is HIV infected at time t**

$E_{t}^{\text{inf}}$indicates whether the subject has a long term (condomless sex) partner who is infected ($E_{t}^{\text{inf}}=1$ if infected, else $E_{t}^{\text{inf}}=0$). A long term partner at time t can be infected either because (i) a new long term partnership has been formed and the partner was already infected, (ii) because a long term partner at t-1, which has remained a long term partner at time t, has become infected, or (iii) because an infected long term partner has remained as a long term partner.

For (i):

$E_{t}^{\text{inf}}=1\text{ if }L_{(t-1)}^{\text{inf}}\geq1$ (i.e. if the subject had a short term partner at time t-1 who was infected then it is assumed that the new long term partner is infected)

For (ii):

The probability that a long term partner of a subject of age group a and gender g becomes infected is derived from the HIV incidence at t-1 for age group a (i.e. the same age group) and gender 1-g, $i_{a(1-g)(t-1)}$ among the sexually active population, either with a long term partner or at least one short term partner (which is given by the number of subjects newly infected in age group at time *t-1* divided by the number of HIV-uninfected subjects in age group at *t-1*, who had condom-less relationships, either long or short term)

$$\left\{ \begin{aligned} E_{t}^{\text{inf}}=1, &U<i_{a\left( 1-g \right)\left( t-1 \right)} \text{where }U \text{randomly sampled from }Uniform(0,1) \\ E_{t}^{\text{inf}}=0, &\text{otherwise} \end{aligned} \right.$$

In order to maintain balance, for each gender, between the number of uninfected people with a long term partner who is infected, and the number of infected people with a long term partner who is uninfected, this incidence $i_{a(1-g)(t-1)}$ is modified at time t dependent on the degree of balance at time t-1.

For (iii):

If $E_{\left( t-1 \right)}^{\text{inf}}=1\text{ and }E_{t}\geq1 \text{then assign }E_{t}^{\text{inf}}=1$

**Determination of the risk of infection from a short term partner**

For each HIV infected short term partner of a subject of gender g and age group a the viral load group, v, of the partner is obtained by sampling from the viral load distribution of those of the opposite gender and specific to the age of the partner. Thus we sample from Uniform(0,1), where the probability of the partner having viral load in group v is given by

$$\frac{\sum_{v} L_{(t-1)}^{\text{inf}}}{\sum L_{(t-1)}^{\text{inf}}}$$

where the numerator is the total number of short-term partnerships had by infected people in viral load group v and the denominator is the total number of short-term partnerships had by infected people (in any viral load group).

Viral load groups are:

(1) < 2.7 log cps/mL

(2) 2.7-3.7 log cps/mL

(3) 3.7-4.7 log cps/mL

(4) 4.7-5.7 log cps/mL

(5) > 5.7 log cps/mL

(6) primary infection.

Once the viral load group, v, of the infected partner is determined, the probability, t_v_, of the subject being infected by the partner is then given according to: t_1_ = Normal (*tr_rate_undetec_vl*,0.000025^2^), t_2_ = Normal (0.01,0.0025^2^), t_3_ = Normal (0.03,0.0075^2^), t_4_ = Normal (0.06,0.015^2^), t_5_ = Normal (0.1,0.025^2^), t_6_ = Normal (*tr_rate_primary*,0.075^2^). These are based on Hollingsworth et al (2008) and Bellan (2015) and are the rates for a longer term partner. The transmission rate for a short term partner is multipled by *fold_tr_newp* (0.35) due to the assumed lower number of sex acts. These probabilities are increased by *fold_change_w*-fold (= 1.5) for female subjects aged > 20, by 2-fold for female subjects aged < 20, and by *fold_change_sti*-fold (= 3.0) if the person has an existing STI (risk of a new STI in any one three month period is given by the number of short term condomless partners / 20 (or 1 if > 20 short term partners)) (Cohen et al 1998, Nicolosia 1994).

We assume that super-infection can occur(i.e. a person can be re-infected with HIV with consequent risk of acquiring new mutations).

Realization of whether the subject is infected by each short term partner is determined by sampling from Uniform(0,1).

**Determination of the risk of infection from a long term partner**

Infected long term partners at time t are classified by whether they are in primary infection (if infection occurred at t-1), whether they are diagnosed with HIV, whether they are on ART, and whether their current viral load is < 2.7 cps/mL or not. The proportion of long term partners with HIV who have HIV diagnosed at time t, $p_{t}^{\text{e,diag}}$, is determined with reference to the difference, $d_{(t-1)}^{\text{e,diag}}$, in the proportion of subjects with HIV who are diagnosed, $\frac{T_{(t-1)}^{\text{diag}}}{T_{(t-1)}^{\text{inf}}}$ and $p_{(t-1)}^{\text{e,diag}}$;

i.e. $d_{(t-1)}^{\text{e,diag}}=\frac{T_{(t-1)}^{\text{diag}}}{T_{(t-1)}^{\text{inf}}}-p_{(t-1)}^{\text{e,diag}}$

where $T_{(t-1)}^{\text{diag}}$ is the total number of subjects diagnosed with HIV at time t-1and $T_{(t-1)}^{\text{inf}}$ is the total number of subjects with HIV (diagnosed and undiagnosed) at time t-1.

$$\left\{ \begin{aligned} \text{if }0<d_{(t-1)}^{\text{e,diag}}\leq0.05 \text{then }p_{t}^{\text{e,diag}}=0.4 \\ \text{if }0.05<d_{\left( t-1 \right)}^{\text{e,diag}}\leq0.10 \text{then }p_{t}^{\text{e,diag}}=0.5 \\ \text{if }0.10<d_{(t-1)}^{\text{e,diag}}\leq0.15 \text{then }p_{t}^{\text{e,diag}}=0.9 \\ \text{if }0.15<d_{(t-1)}^{\text{e,diag}} \text{then }p_{t}^{\text{e,diag}}=0.95 \end{aligned} \right.$$

The proportion of those diagnosed who are on ART, and the proportion of those on ART who have viral load < 2.7 log cps/mL are determined in a similar manner. In this way the proportions diagnosed with HIV, on ART, and with current viral load is < 2.7 log cps/mL are kept similar for the long term partners as in the simulated subjects themselves.

Risk of infection from a long term infected partner is determined by Normal (*tr_rate_primary*, 0.075^2^) if the existing partner is in primary infection (ie. infected at t-1), Normal (*tr_rate_undetec_vl*, 0.000025^2^) if the existing partner has viral load < 2.7 log cps/mL, and Normal (0.05, 0.0125^2^) otherwise.

**4. Natural history of HIV infection**

Figure S2 gives an overview of the modelling of HIV natural history. The model of the natural history of HIV and the effect of antiretroviral therapy has been derived previously and compared with a range of observed data (see Phillips et al Lancet 2008, AIDS 2011, Nakagawa et al 2012, 2015 and associated supplementary material). Below we set out the structure of the model and explain what parameters represent.

**Figure S2.** Overview of modelling of natural history of HIV infection.


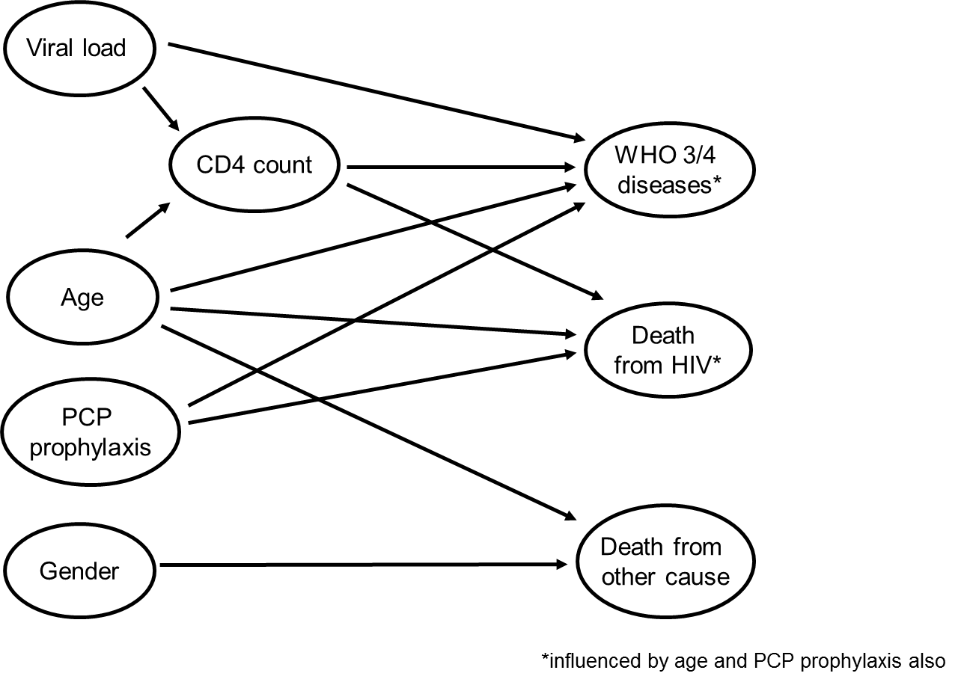


**Determination of changes in viral load and CD4 count**

**Initial log_10_ viral load** (V_set_) is sampled from Normal(4.0,0.5^2^)

This viral load (V_set_) is assumed to be that reached after primary infection. It is not used to determine the risk of transmission in primary infection itself.

**Initial CD4 count**, modelled on the square root scale, is partially dependent on initial viral load and given by

Square root CD4 count = *mean_sqrtcd4_inf* (= 27.5) - (1.5 x V_set_) + Normal(0,2^2^) – ((age – 35) x 0.05)

Initial virus is assumed to be R5-tropic. Shift to presence of X4 virus is assumed to depend on viral load. Probability of a shift per 3 months is given by 10^v^ x 0.0000004, where v is the current log_10_ viral load.

Viral load change (vc) from period t-1 to period t (i.e. in 3 months) is given by

vc(t-1( = (gx x 0.02275 + Normal(0, 0.05^2^) + ((age(t-1) - 35) x 0.00075)

*gx*=1 viral load at t (v(t)) = v(t-1)+ vc(t-1)

CD4 count changes from period t-1 to t are dependent on the current viral load (i.e. viral load at time t-1) and are given by sampling from a Normal distribution with standard deviation *sd_cd4* and mean *fx* (= 1.0) times the values as follows:

Viral load Change in

at t-1 square root

CD4 count

(per 3 mths)

-----------------------------------------------

< 3.0 +0.000

3-0 +0.022

3.5- +0.085

4.0- - 0.400

4.5- - 0.400

5.0- - 0.850

5.5- - 1.300

6.0- - 1.750

-----------------------------------------------

The change additionally is affected by the current age as follows:

People with X4 virus present experience an additional change in square root CD4 count of -0.25.

These estimates were derived based on consideration of evidence from natural history studies (Pantazis 2005, Sabin JAIDS 2000, Hubert J-B 2000, O'Brien 1998, Henrard 1995, Lyles 2000, Touloumi 2004, Mellors 1997, Koot 1993) and were selected in conjunction with other relevant parameter values to provide a good fit to the incubation period distribution. Differences that have been found in initial viral load by sex, age and risk group are not currently incorporated in the model.

**Risk of clinical disease and death in HIV infected people**

**Occurrence of WHO 4 diseases**

The rate of WHO 4 diseases according to CD4 count per 3 months is given below.

**Table S7.** Rate of WHO stage 4 disease according to CD4 count and viral load.

| If cd4 > 650 | rate=0.002 | if 500 < cd4 < 650 | rate=0.010 |
| --- | --- | --- | --- |
| if 450 < cd4 < 500 | rate=0.013 | if 400 < cd4 < 450 | rate=0.016 |
| if 375 < cd4 < 400 | rate=0.020 | if 350 < cd4 < 375 | rate=0.022 |
| if 325 < cd4 < 350 | rate=0.025 | if 300 < cd4 < 325 | rate=0.030 |
| if 275 < cd4 < 300 | rate=0.037 | if 250 < cd4 < 275 | rate=0.045 |
| if 225 < cd4 < 250 | rate=0.055 | if 200 < cd4 < 225 | rate=0.065 |
| if 175 < cd4 < 200 | rate=0.080 | if 150 < cd4 < 175 | rate=0.10 |
| if 125 < cd4 < 150 | rate=0.13 | if 100 < cd4 < 125 | rate=0.17 |
| if 90 < cd4 < 100 | rate=0.20 | if 80 < cd4 < 90 | rate=0.23 |
| if 70 < cd4 < 80 | rate=0.28 | if 60 < cd4 < 70 | rate=0.32 |
| if 50 < cd4 < 60 | rate=0.40 | if 40 < cd4 < 50 | rate=0.50 |
| if 30 < cd4 < 40 | rate=0.80 | if 20 < cd4 < 30 | rate=1.10 |
| if 10 < cd4 < 20 | rate=1.80 | if 0 < cd4 < 10 | rate=2.50 |
|  |  |  |  |
| Independent effect of viral load | |  |  |
| if v < 3 | rate = rate x 0.2 |  |  |
| if 3 <= v < 4 | rate = rate x 0.3 |  |  |
| if 4 <= v < 4.5 | rate = rate x 0.6 |  |  |
| if 4.5 <= v < 5 | rate = rate x 0.9 |  |  |
| if 5 <= v < 5.5 | rate = rate x 1.2 |  |  |
| if 5.5 <= v | rate = rate x 1.6 |  |  |

This is informed by (Cascade Collaboration 2003) .

**Independent effect of age**

rate = rate x (age / 38)^1.2^

**Table S8.** Example model outputs of incubation period by age. Kaplan-Meier percent with WHO 4 Event. Compare with Darby et al 1996. This varies by model run due to the sampling of the value of the parameter *fx d*escribed above.

Age at infection Years from infection

1 3 5 10 15 20

------------------------------------------------------------------------------------------------

15- 0.6% 4% 14% 50% 75% 89%

25- 1.1% 2% 23% 67% 88% 97%

35- 2.1% 13% 34% 82% 97% 100%

45- 3.7% 21% 54% 93% 100% 100%

55- 1.4% 24% 59% 96% 100% 100%

------------------------------------------------------------------------------------------------

**5. HIV testing and diagnosis of HIV infection**

HIV testing was assumed introduced in 1996. At that time we assumed 20% of the population were resistant to be tested for HIV unless symptomatic and this decreased linearly to 5% by the end of 2010. In the model this group has no possibility of getting tested for HIV unless symptomatic. Limited data are available to inform this parameter (proxy variables are the proportion who reported never being tested for HIV and, more precisely, the proportion who refuse HIV testing), nevertheless we considered it important to take this into account, given the evidence that not everyone accepts HIV testing for various reasons. The level of acceptability of provider initiated HIV testing and counselling (PITC) in resource limited settings is extremely variable from levels of 99%, observed in inpatients in Uganda (Wanyenze 2011) to 31% among outpatients in South Africa (Bassett 2007). Among pregnant women the level of acceptability of PITC seems to be higher, varying from 76 to 99.9% (Hensen 2012), while the estimated acceptability of home-based counselling and testing has been estimated in a meta-analysis to be 83% (Sabapathy 2012). This variability seems to be related mainly to the quality of the intervention delivered and calendar time. Acceptability seems to have increased over time due to the reduction in stigma and higher availability of ART, therefore we thought it was reasonable to assume a decline in the proportion resistant to be tested for HIV down to 5% in 2010.

For the remainder of the population (non-resistant to HIV testing), increasing gender and age-specific rates of HIV testing (for the 1st time and for repeat testing) since 1996 were assumed (parameter *an_lin_incr_test* sampled at the start of each model run), to reflect the range of levels of testing observed in Malawi and other countries in the region. This increase in testing is assumed to stop so that testing rates reach a plateau (*date_test_rate_plateau*). We assume some targeting of testing such that those having a condomless sex partner since last test are more likely to test – the degree of such targeting is conveyed by the parameter *test_targeting*. Pregnant women experience an additional probability of being tested in the ANC, which increases over calendar time (*rate_testanc_inc*).

People with acute symptoms (WHO stage 4, 3 or active TB) are assumed to have a higher chance of testing for HIV in that 3 month period and a higher chance of being linked to care once diagnosed and the increase over time in this testing probability *incr_test_rate_sympt*. Like all other parameters mentioned in this section this is sampled at the start of each model run.

**5. Modelling the effect of ART**

This is described in detail in the Appendix to a recent publication (Phillips et al, 2019).

**6. Prior distributions for parameters**

**Table S9**

| Parameter name | Description* | Distribution |
| --- | --- | --- |
| ***Parameters relating to sexual behaviour*** | | |
| *swn* | Value of multiplicative factor determining numbers of partners for those in highest new partner group | Uniform(4,25) |
| *highsa* | Value of and fold change in multiplicative factor determining numbers of partners for those in second highest new partner group | Uniform(3,10) |
| *p_rred_p* | Indicates the proportion of the population in whom the sexual risk behaviour is very low | Uniform(0.1,0.5) |
| *p_hsb_p* | Indicates the proportion of the population in whom the sexual risk behaviour has a tendency to be higher than average | Uniform(0,0.2) |
| *newp_factor* | Overall average level of sexual risk behaviour. The correlation with the above parameters induced by the sampling of this parameter is to provide a focus on parameter space most likely to give low values of the overall fit. For example, if the sampling of *swn* and *highsa* give values at the high end of the distribution and sampling of *p_rred_p* produces a value at the low end then the model simulation run will produce an epidemic which is too large, unless there is some compensation when selecting the value of this parameter. | 6.5/highsa x 14.5/swn x p_rred_p/0.25 x 0.1/p_hsb_p x logNormal(0, 0.5^2^) |
| *conc_ep* | Parameter indicating the degree to which those with a long term condomless sex partner have a lower of higher probability of short term condomless sex partners than those without a long term condomless sex partner. | LogNormal(0,0.6) |
| *ych_risk_beh_newp* | Degree of reduction in condomless sex with short term partners per year from 1995 – 2000 | Uniform(0.12,0.20) |
| *ych_risk_beh_ep* | Degree of reduction in condomless sex per year with long term partners from 1995-2000 | Uniform(0,0.08) |
| *ch_risk_diag_newp* | Degree of reduction in condomless sex with short term partners in a person diagnosed with HIV | Uniform(0.7,1.0) |
| *ch_risk_diag* | Degree of reduction in condomless sex with long term partner in a person diagnosed with HIV | Uniform(0.7,1.0) |
| ych2_risk_beh_newp | Degree of change in condomless sex with short term partners per year from 2010 – 2015 | Uniform(-0.04,0.04) |
| sex_beh_trans_matrix | Matrix indicating transition probabilities between categories of new partner levels. | Uniform sampling from matrices 1-9, independently for males and females. (Table S3) |
| **Parameter relating to transmission** | | |
| *res_trans_factor* | parameter determining the probability that if resistance mutation present in source partner that this is not present/detectable in virus new host | 0.67: 20%  0.80: 15%  1.00: 30%  1.25: 15%  1.50: 10%  2.00: 10% |
| **Parameters relating to HIV testing** | | |
| *an_lin_incr_test* | Parameter determining the rate of increase in HIV testing (any testing outside ANC) | Uniform(0.0005,0.0085) |
| *rate_testanc_inc* | Rate of increase in testing in ANC clinics | Uniform(0.02,0.06) |
| *test_targeting* | Degree to which testing is targeted to peoplewho have had condomless sex with > 1 new partner since last test. 1 means no targeting, 4 means rate is 4 times higher in this group than in other adults. | Uniform(1,4) |
| *incr_test_rate_sympt* | The rate of increase over time in the probability of a person with a WHO stage 3 or 4 disease is tested for HIV. | Uniform(1.02,1.25) |
| **Parameters relating to pre-ART care and progression of HIV** | | |
| *fx* | Multiplicative factor to alter the average rate of CD4 count decline in natural HIV progression (which thus alters the incubation period distribution). | Uniform(0.7,1.1) |
| *prob_loss_at_diag* | Probability that a person is immediately lost after initial HIV diagnosis. | Uniform(0.1,0.4) |
| *rate_lost* | For people under care yet to start ART or previously have taken ART, the rate of being lost to care per 3 mths. | Uniform(0.05,0.25) |
| *rate_return* | Probability of return to care for a person who has been diagnosed with HIV (and may have started ART) but is now lost and not on ART, without current WHO stage 3 or 4 disease, per 3 months. | Uniform(0.0,0.2) |
| *prob_return_adc* | Probability of return to care for a person who has been diagnosed with HIV (and may have started ART) but is now lost and not on ART and has a WHO stage 4 condition. This is a probability that operates just for the 3 month period that the events occurs. | Uniform(0.4,0.8) |
| *rate_loss_persistence* | Rate of loss from majority virus of transmitted resistance mutations (per 3 months) | 0.000: 10%  0.005: 10%  0.010: 10%  0.015: 40%  0.020: 30% |
| **Parameters relating to people on ART** | | |
| *adh_pattern* | Population adherence profile; described in terms of the proportion having a given average adherence and period-to-period variability in adherence. | Three alternative population adherence profiles |
| *pr_art_init* | Probability of ART initiation in a person in care who is eligible according to current criteria. | Uniform(0.05,0.85) |
| *prob_lost_art* | For a person who interrupts / stops ART the probability that they are simultaneously lost from care. | Uniform(0.50,0.95) |
| *rate_restart* | Rate of restart of ART for people who previously have been on ART and have returned to care, per 3 months. | Uniform(0.2,0.8) |
| *rate_int_choice* | Rate of interruption / stopping of ART per 3 months. Also influenced by current drug toxicity and underlying tendency to adhere. | Uniform(0.005,0.040) |
| *incr_rate_int_low_adh* | Parameter indicating the extent to which people with a long term average adherence in the lowest group have a multiplicatively increased risk of ART interruption. | Uniform(1,2) |
| *pr_switch_line* | Probability of switch to second line per 3 months in a person who has fulfilled the failure criteria for first line failure. | Uniform(0.05,0.20) |
| *clinic_not_aw_int_frac* | If a person interrupts ART, the probability that this is not disclosed to the clinic and they are classified as being on ART | Uniform(0.1,0.9) |

* parameters are also described in the Modelling Details section above.

Setting scenarios in which the HIV prevalence in 2017 was below 3% or above 35% were not included (or the relative prevalence in 25-35 year old women was less than 1.5 times greater than that in 25-35 year old men), and the same was the case for setting scenarios in which the probability of an HIV test when HIV-related symptoms are present is below 0.30 in 2017, or in which the number of condomless sex sex workers was below 2,500 or above 200,000.

**7. Costs and disability weights**

**Table S10** **Unit Costs and disability weights for DALYs**

| **Item** | **Unit Cost** | **Source / explanation** |
| --- | --- | --- |
| Drug costs per year (including supply chain):    First-line: tenofovir/3TC/efavirenz  First-line: tenofovir/3TC/dolutegravir  Second-line: zidovudine/3TC/atazanavir | $120 ($100 without supply chain costs)  $90 ($75 without supply chain costs)  $343 ($286 without supply chain costs) | Untangling the web of antiretroviral price reductions. 18th Edition – July 2016. [www.msfaccess.org](http://www.msfaccess.org).  CHAI. ARV Market Report. The state of the antiretroviral drug market in low- and middle-income countries, 2016-202. Issue 8, September 2017  <https://clintonhealthaccess.org/2017-arv-market-report/>  <http://www.who.int/hiv/mediacentre/news/high-quality-arv-reduced-price/en/> |
| Cost of treatment of a WHO stage 4 condition over 3 months (cost is incurred for 3 months)  Cost of treatment of a WHO stage 3 condition over 3 months (cost is incurred for 3 months)  Cost of treatment of TB per 3 months (cost is incurred for 6 months)  Cotrimoxazole annual cost | $200  $20  $50  $5 | Specific data not available on average unit costs of treating WHO stage 3 and 4 conditions and per clinic visit costs - costs used are informed by evidence synthesis from studies that cost according to current CD4 count of those in pre-ART care, cost of ART initiation, which also include costs of CD4 tests (Eaton J et al. Health benefits, costs, and cost-effectiveness of earlier eligibility for adult antiretroviral therapy and expanded treatment coverage: a combined analysis of 12 mathematical models. Lancet Global Health 2014: E23-E34) |
| CD4 count measurement | $10 | Hyle, E. P., Jani, I. V, Lehe, J., Su, A. E., Wood, R., Quevedo, J., … Walensky, R. P. (2014). The Clinical and Economic Impact of Point-of-Care CD4 Testing in Mozambique and Other Resource-Limited Settings: A Cost-Effectiveness Analysis. PLoS Med, 11(9), e1001725. doi:10.1371/journal.pmed.1001725. Keebler D, Revill P, et al. How Should HIV Programmes Monitor Adults on ART? A Combined Analysis of Three Mathematical Models. Lancet Global Health 2014. E35-E43. |
| Viral load measurement: | $22 | Human resource costs $3, sample collection consumables $2, relaying of results $2 (this costing information was provided by Medecin Sans Frontiers (MSF) (K Bonner), update February 2014), running the test (including equipment and other costs such as consumables, maintenance and shipping) $15 (http://www.theglobalfund.org/en/mediacenter/newsreleases/2015-06-10_New_Approach_on_HIV_Viral_Load_Testing/ http://www.theglobalfund.org/en/procurement/viral-load-early-infant-diagnostics/) |
| Non-ART programme costs per year, $40 per year if on tiered care due to viral load < 1000 | $80 | Tagar E, Sundaram M, Condliffe K, Matatiyo B, Chimbwandira F, et al. (2014) Multi-Country Analysis of Treatment Costs for HIV/AIDS (MATCH): Facility-Level ART Unit Cost Analysis in Ethiopia, Malawi, Rwanda, South Africa and Zambia. PLoS ONE 9(11): e108304. doi:10.1371/journal.pone.0108304; |
| Cost of the targeted adherence counselling intervention triggered by a viral load > 1000 copies/mL | $10 | Assumption |
| HIV test (including personnel costs) | $3.70 | Personal Communication, CHAI (Nov 2016; [labservices@clintonhealthaccess.org](mailto:labservices@clintonhealthaccess.org). |
|  |  |  |

**Table S11. Disability weights** (informed by Salomon et al*)

Values are 1 except for the following:

| **Condition in current 3 month period** | **Disability weight for current 3 month period** |
| --- | --- |
| Any drug toxicity in current 3-month period | 0.95 |
| Any WHO stage 3 condition (except TB) in current 3-month period | 0.78 |
| TB in current 3-month period | 0.60 |
| Any WHO stage 4 condition in current 3-month period | 0.46 |

* Salomon JA, Vos T, Hogan DR, et al. Common values in assessing health outcomes from disease and injury: disability weights measurement study for the Global Burden of Disease Study 2010. Lancet 2012; 380: 2129–43.

**References**

Bassett, I. V. et al. Routine voluntary HIV testing in Durban, South Africa: the experience from an outpatient department. Journal of acquired immune deficiency syndromes (1999) 46, 181 (2007)

Bellan SE, Dushoff J, Galvani AP, Meyers LA (2015) Reassessment of HIV-1 Acute Phase Infectivity: Accounting for Heterogeneity and Study Design with Simulated Cohorts. PLoS Med 12(3): e1001801. doi:10.1371/journal.pmed.1001801

Cambiano, V., Bertagnolio, S., Jordan, M. R., Lundgren, J. D. & Phillips, A. Transmission of drug resistant HIV and its potential impact on mortality and treatment outcomes in resource-limited settings. Journal of Infectious Diseases 207, S57-S62 (2013).

Cambiano, V. et al. Predicted levels of HIV drug resistance: potential impact of expanding diagnosis, retention, and eligibility criteria for antiretroviral therapy initiation. Aids 28, S15-S23 (2014).

Cambiano, V. et al. Assessment of the potential impact and cost-effectiveness of self-testing for HIV in low-income countries. Journal of Infectious Diseases, jiv040 (2015).

CASCADE Collaboration. Short-term risk of AIDS according to current CD4 cell count and viral load in antiretroviral drug-naïve individuals and those treated in the monotherapy era. *AIDS*, 2004. 18(1):51-8.

Cohen, M. S. Sexually transmitted diseases enhance HIV transmission: no longer a hypothesis. The Lancet 351, S5-S7 (1998).

Darby SC, Ewart DW, Giangrande PLF, et al. Importance of age at infection with HIV-1 for survival and development of AIDS in UK haemophilia population. Lancet 1996; 347: 1573–79.

Gregson, S., Zhuwau, T., Ndlovu, J. & Nyamukapa, C. A. Methods to reduce social desirability bias in sex surveys in low-development settings: experience in Zimbabwe. Sexually transmitted diseases 29, 568-575 (2002).

Gregson, S. et al. HIV decline in Zimbabwe due to reductions in risky sex? Evidence from a comprehensive epidemiological review. International Journal of Epidemiology, dyq055 (2010).

Halperin, D. T. et al. A surprising prevention success: why did the HIV epidemic decline in Zimbabwe. PLoS medicine 8, e1000414 (2011).

Hollingsworth, T. D., Anderson, R. M. & Fraser, C. HIV-1 transmission, by stage of infection. Journal of Infectious Diseases 198, 687-693 (2008).

Johnson, L. F., Dorrington, R. E., Bradshaw, D., Pillay-Van Wyk, V. & Rehle, T. M. Sexual behaviour patterns in South Africa and their association with the spread of HIV: insights from a mathematical model. Demographic Research 21, 289 (2009).

Nakagawa, F. et al. Projected life expectancy of people with HIV according to timing of diagnosis. Aids 26, 335-343 (2012).

Nakagawa, F. et al. Projected lifetime healthcare costs associated with HIV infection. (2015).

Nicolosi, A. et al. The Efficiency of Male-to Female and Female-to-Male Sexual Transmission of the Human Immunodeficiency Virus: A Study of 730 Stable Couples. Epidemiology 5, 570-575 (1994).

Henrard, D. R. et al. Natural history of HIV-1 cell-free viremia. Jama 274, 554-558 (1995).

Hensen, B. et al. Universal voluntary HIV testing in antenatal care settings: a review of the contribution of provider‐initiated testing & counselling. Tropical Medicine & International Health 17, 59-70 (2012).

Hubert, J.-B. et al. Natural history of serum HIV-1 RNA levels in 330 patients with a known date of infection. Aids 14, 123-131 (2000).

Koot, M. et al. Prognostic value of HIV-1 syncytium-inducing phenotype for rate of CD4+ cell depletion and progression to AIDS. Annals of internal medicine 118, 681-688 (1993).

Lyles, R. H. et al. Natural history of human immunodeficiency virus type 1 viremia after seroconversion and proximal to AIDS in a large cohort of homosexual men. Journal of Infectious Diseases 181, 872-880 (2000).

Mellors, J. W. et al. Plasma viral load and CD4+ lymphocytes as prognostic markers of HIV-1 infection. Annals of internal medicine 126, 946-954 (1997).

O'Brien, T. R., Rosenberg, P. S., Yellin, F. & Goedert, J. J. Longitudinal HIV-1 RNA levels in a cohort of homosexual men. JAIDS Journal of Acquired Immune Deficiency Syndromes 18, 155-161 (1998).

Pantazis, N., Touloumi, G., Walker, A. & Babiker, A. Bivariate modelling of longitudinal measurements of two human immunodeficiency type 1 disease progression markers in the presence of informative drop‐outs. Journal of the Royal Statistical Society: Series C (Applied Statistics) 54, 405-423 (2005).

Phillips, A. N. et al. Outcomes from monitoring of patients on antiretroviral therapy in resource-limited settings with viral load, CD4 cell count, or clinical observation alone: a computer simulation model. The Lancet 371, 1443-1451 (2008).

Phillips, A. N. et al. Effect on transmission of HIV-1 resistance of timing of implementation of viral load monitoring to determine switches from first to second-line antiretroviral regimens in resource-limited settings. Aids 25, 843-850 (2011).

Phillips AN, Cambiano V, Miners A, Revill P, Pillay D, Lundgren JD, et al. Effectiveness and cost-effectiveness of potential responses to future high levels of transmitted HIV drug resistance in antiretroviral drug-naive populations beginning treatment: modelling study and economic analysis. Lancet HIV 2014 October 14, 2014

Phillips AN, Stover J, Cambiano V, Nakagawa V, Jordan MR, Pillay D, et al. Impact of HIV drug resistance on HIV/AIDS associated mortality, new infections and antiretroviral therapy program costs in sub-Saharan Africa. J Infect Dis 2017 DOI: 10.1093/infdis/jix089

Phillips AN, Cambiano V, Nakagawa F, Revill P, Jordan MR, Hallett TB et al. Cost-effectiveness of public-health policy options in the presence of pretreatment NNRTI drug resistance in sub-Saharan Africa: a modelling study Lancet HIV 2018; 5: E146-E154 DOI: 10.1016/S2352-3018(17)30190-X

Phillips AN, Venter F, Havlir D, Pozniak A, Kuritzkes D, Wensing et al. Risks and benefits for use of dolutegravir-based antiretroviral drug regimens in sub Saharan Africa: a modelling study. Lancet HIV 2019 (in press).

Sabapathy, K., Van den Bergh, R., Fidler, S., Hayes, R. & Ford, N. Uptake of home-based voluntary HIV testing in sub-Saharan Africa: a systematic review and meta-analysis. (2012).

Sabin, C. A. et al. Course of viral load throughout HIV-1 infection. JAIDS Journal of Acquired Immune Deficiency Syndromes 23, 172-177 (2000).

Touloumi, G. et al. Differences in HIV RNA levels before the initiation of antiretroviral therapy among 1864 individuals with known HIV-1 seroconversion dates. Aids 18, 1697-1705 (2004).
